# Improving documentation of clinical care within a clinical information network: an essential initial step in efforts to understand and improve care in Kenyan hospitals

**DOI:** 10.1136/bmjgh-2016-000028

**Published:** 2016-05-24

**Authors:** Timothy Tuti, Michael Bitok, Lucas Malla, Chris Paton, Naomi Muinga, David Gathara, Susan Gachau, George Mbevi, Wycliffe Nyachiro, Morris Ogero, Thomas Julius, Grace Irimu, Mike English

**Affiliations:** 1 Health Services Unit, KEMRI-Wellcome Trust Research Programme, Nairobi, Kenya; 2 Nuffield Department of Medicine, University of Oxford, Oxford, UK; 3 College of Health Sciences, University of Nairobi, Nairobi, Kenya; 4 Ministry of Health, Government of Kenya, Nairobi, Kenya

## Abstract

In many low income countries health information systems are poorly equipped to provide detailed information on hospital care and outcomes. Information is thus rarely used to support practice improvement. We describe efforts to tackle this challenge and to foster learning concerning collection and use of information. This could improve hospital services in Kenya.

We are developing a Clinical Information Network, a collaboration spanning 14 hospitals, policy makers and researchers with the goal of improving information available on the quality of inpatient paediatric care across common childhood illnesses in Kenya. Standardised data from hospitals' paediatric wards are collected using non-commercial and open source tools. We have implemented procedures for promoting data quality which are performed prior to a process of semi-automated analysis and routine report generation for hospitals in the network.

In the first phase of the Clinical Information Network, we collected data on over 65 000 admission episodes. Despite clinicians' initial unfamiliarity with routine performance reporting, we found that, as an initial focus, both engaging with each hospital and providing them information helped improve the quality of data and therefore reports. The process has involved mutual learning and building of trust in the data and should provide the basis for collaborative efforts to improve care, to understand patient outcome, and to evaluate interventions through shared learning.

We have found that hospitals are willing to support the development of a clinically focused but geographically dispersed Clinical Information Network in a low-income setting. Such networks show considerable promise as platforms for collaborative efforts to improve care, to provide better information for decision making, and to enable locally relevant research.

**Key questions**

**What is already known about this topic?**

- Collaborative health information networks have helped improve outcomes of care, accelerated knowledge discovery and advanced cross-domain development of digital architecture to support research in high-income settings. Central to such networks is the collection of standardised data across hospital sites that can be used for tracking or benchmarking performance while promoting the sharing of experiences and innovations to improve care.

**What are the new findings?**

- Establishing health information networks in low-resource settings has multiple unique challenges that new research needs to address. These challenges include the development of new data collection procedures and new methods to implement the provision of accurate reporting to hospitals.

**Recommendations for policy**

- This study provides evidence that operationalising clinical information networks in low-income countries can be achieved by addressing:
  – Technical rules for improving the data quality collected in a resource-limited setting using open source and non-commercial standardised patient data collection tools.
  – Behavioural rules of collaborative health networks to improve organisational culture to enable new systems for gathering and using information for improving care delivery.

## Introduction

The need to improve healthcare delivery has been highlighted in a number of reports from low and middle-income countries (LMIC),^1^ ^2^ including Kenya.^3–5^

The Kenya Medical Research Institute (KEMRI)-Wellcome Trust Research Programme's (KWTRP) Health Services Unit has collaborated with the Kenyan Ministry of Health since 2002 to develop national evidence-based clinical guidelines for paediatric care,^6^ to conduct implementation research and pragmatic clinical trials,^7^ ^8^ and to conduct surveys of the quality of care within hospitals.^9^ On the basis of these experiences and a review of the wider literature,^10^ a new programme of work was developed to focus on improving the delivery of essential interventions during inpatient paediatric care.

Kenya is similar to many LMIC in that hospitals often have no electronic systems for recording the care they provide. This means that in order to improve the delivery of essential interventions, we first need to establish a new method for collecting data on paediatric admissions to Kenyan hospitals. A new partnership between researchers, the Ministry of Health, The Kenyan Paediatric Associated and 14 country (district) level hospitals was formed to create a Clinical Information Network (CIN) to provide an accurate picture of healthcare provision to paediatric inpatients in the participating hospitals.

The CIN follows the approach of other clinical networks that have been a feature of efforts to improve care in high-income (eg, the Northern Neonatal Network,^11^ the Vermont Oxford Network^12^) and middle-income (eg, the Child Healthcare Problem Identification Programme^13^) countries. A network has been described as ‘a grouping that aims to improve clinical care and service delivery using a collegial approach to identify and implement a range of [improvement] strategies’,^14^ and the CIN follows this approach.

More recently, clinical information networks have helped improve outcomes of care,^15^ accelerated knowledge discovery,^16^ and advanced cross-domain development of digital architecture to support research.^17^ Central to such networks is the collection of standardised data across sites that can be used for tracking or benchmarking performance while promoting the sharing of experiences and innovations to improve care. However, there are few published reports of attempts to develop collaborative information networks in LMIC.

In this paper, we describe the challenges faced by Kenya and other low-income countries with the collection of data on routine care and provide an overview of the approach used to address these challenges in the area of paediatric admissions, the focus of our CIN. We describe how hospitals were provided with routine reports to help improve clinical documentation, and then consider the potential future value of such a network.

## Background

Quality is multidimensional and often described as comprising structure (inputs), process (activities) and outcomes.^18–20^ In recent years, increasing attention has been devoted to assessing the process aspect of delivering quality in healthcare. Optimal processes can be defined by clinical practice standards or summarised as guidelines. These can provide an explicit link between research evidence and practice. It therefore follows that the gap between these standards and the care that is actually delivered provides one measure of quality care: it indicates how successfully (new) interventions are adopted in practice and also whether any benefits from research are realised. Central to many strategies to improve process quality is therefore the ability to measure adherence to guidelines and tracking the progress of such indicators as part of ‘Plan, Do, Study Act’ cycles. However, in low-income settings, routine health information systems often provide data of poor quality,^4^ ^21^ which preclude their use in such improvement exercises. Specific challenges are listed in the following section.

### The challenges

#### Poor clinical documentation

Inpatient clerking in public district hospitals in LMIC is predominantly paper-based and patients' clinical features are often poorly documented.^22^ This often makes the subsequent medical records an inadequate source of accurate patient data. Information on patient assessment, investigations carried out and treatment prescribed are also often only partially documented. However, in prior work, the CIN team has been able to develop and implement a medical record tool that enables clinicians to document patient admissions in a standardised fashion, and data on treatments can also be improved through the use of routine treatment charts.^7^ ^23^

#### Limitations of National Health Information Systems

Kenya has an electronic national health data collection system, called DHIS2, that is now in use in many low-income countries.^24^ Summary data from hospitals are usually collated from paper medical records (which suffer from the issues described above) and entered through a web-portal onto the national DHIS2 system. In current practice, each disease episode is assigned an International Classification of Diseases 10th Edition code and DHIS summary reports are based on these codes rather than on patient counts.^25^ As a result, a patient with more than one diagnosis contributes more than one disease episode and this makes it hard to disambiguate prevalence rates from DHIS reports by patient count rather than disease episode count. Use of these limited data for basic tasks (eg, tracking patient outcomes) is further hampered by poor standardisation of coding and gaps in reporting data such as whether the patient lived or died.^5^ ^22^ The lack of information on patients' key symptoms or signs, any investigations used and their results and of how treatments are used makes exploring the process aspects of quality impossible using data collected through the current national Health Information System.

#### Information culture in hospitals

Kenyan hospitals often do not have a culture of using information to systematically improve patient care as the lack of longitudinal data (as described above) means that information is not available to inform efforts at quality improvement audit cycles.^26^ ^27^ Some sporadic information-gathering exercises are conducted, such as mortality audits, and most health institutions have a process for delivering Continuous Medical Education (CME) to physicians. However, these exercises rarely feed back into process improvement due to the insufficiency and poor quality of the available information, and a lack of subsequent monitoring or evaluation of any possible change in care.^22^

The CIN therefore initially set out to overcome these challenges and produce high-quality process and outcome data from individual admissions to paediatric wards in Kenyan hospitals as a prelude to using these data to inform improvement strategies. Our initial focus was on improving information on the most common childhood illnesses in Kenya, which account for up to 80% of all admission episodes in many African countries and the CIN.^28^ Quality of care indicators for these common illnesses have previously been identified through an international and national Delphi exercise linked to standards encompassed in the WHO and Kenyan paediatric guidelines.^29^ ^30^ These indicators have been successfully used in previous assessments of the quality of paediatric inpatient care.^3–5^ ^9^ ^31^ Our strategies for tackling the challenges of enabling routine measurement of such quality indicators are outlined in the next section.

## Data quality improvement strategies

### Improving routine clinical documentation

To facilitate improved clinical documentation, hospitals were encouraged to promote good prescribing practices and to implement both more formal discharge forms and a standard paediatric admission record.^23^ Much of the focus of initial data use was to provide feedback to hospitals on the quality of their clinical documentation. This anticipates improvements from network activities, which have included feedback and mentorship through telephone calls and 4 monthly face-to-face meetings.^32^ ^33^

### The informatics framework

**Figure 1:**
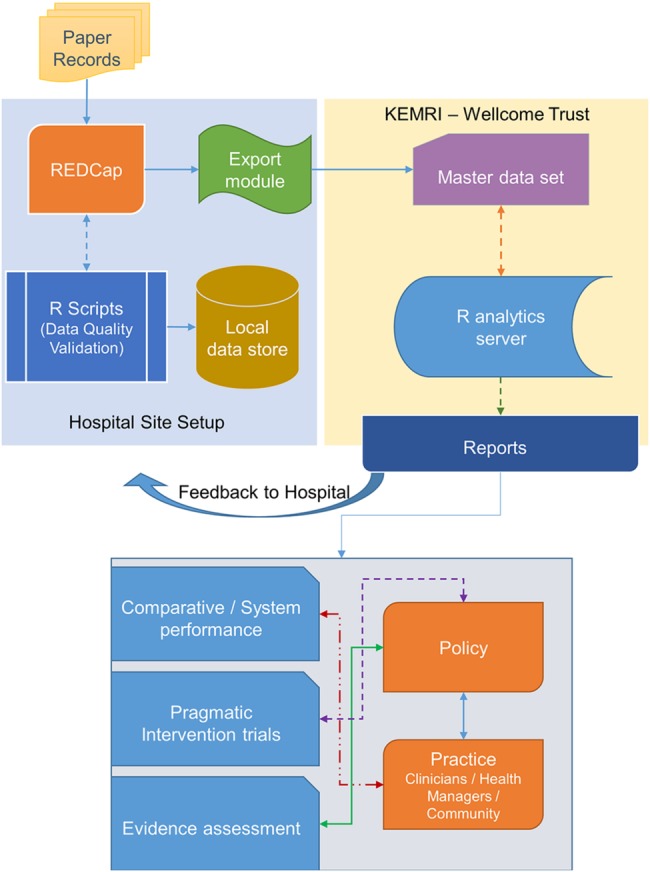
Informatics infrastructure framework to support data use. KEMRI, Kenya Medical Research Institute.

Data capture in CIN hospitals happens at the point of patient discharge where data from the paediatric inpatient paper records are abstracted directly into a non-commercial electronic tool, REDCap.^34^ A minimum data set required for the national reporting system (DHISv2^24^) is collected on all patients admitted to the paediatric wards for all sites. Comprehensive data for all admissions aged 1 month or more without burns or a surgical diagnosis to the paediatric ward(s) are entered in 12 hospitals and, because of the high workload, on a random selection of records in 2 hospitals (35% and 70% records). The comprehensive data comprise clinical, investigation and treatment data focused on admission events and then discharge data with up to 350 variables per patient encounter. As is summarised in figure 1, data are collected by trained clerks^35^ and preprogrammed field validation rules in the REDCap tool are used to check data quality as it is entered. All data subsequently shared with the central network analysis team are de-identified. R (R Core Team, R: *A Language and Environment for Statistical Computing*. 2014: Vienna, Austria) statistical software has been installed on hospital sites' computers and, through a process of meta-programming (writing code that writes itself during runtime based on predefined clinical guidelines^22^ ^23^ ^29^), R software autogenerates code that is used for running on-site checks daily. It then also cleans and recodes data to enable indicator measurement and reporting. These R resources are available for reuse in other projects.^36^ ^36^ A detailed report of CIN's data management framework is described elsewhere.^35^

## Results of assessment

### Data use

The auto-generated R scripts are used to prepare reports for each hospital on a 2–3 monthly basis. Additionally, a combined hospital report is generated for the Ministry of Health in Kenya using cleaned datasets from all hospitals. For each hospital, data were initially used to provide feedback on the completeness of documentation of a set of 16 core symptoms and signs on admission. Over the first 2 years of operation, reports have been delivered to CIN hospitals on eight occasions. There have also been three face-to-face CIN meetings with paediatricians that included, on two occasions, senior nurses and health record offices. These reports and meetings were supplemented with telephone discussions with paediatricians every 2–4 weeks that promoted better use of the paediatric admission record and documentation of a wider range of clinical and demographic data (n=49 demographic, symptom and sign characteristics). To promote informal benchmarking, the adequacy of documentation for the core 16 clinical variables was also summarised and presented in reports that span all hospitals.

**Figure 2:**
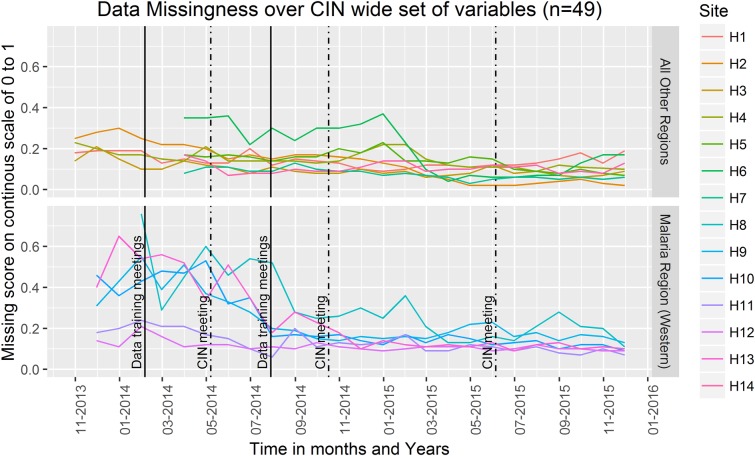
Trends of rate of missing data for all core signs and symptoms documented during admission. CIN, Clinical Information Network.

**Figure 3:**
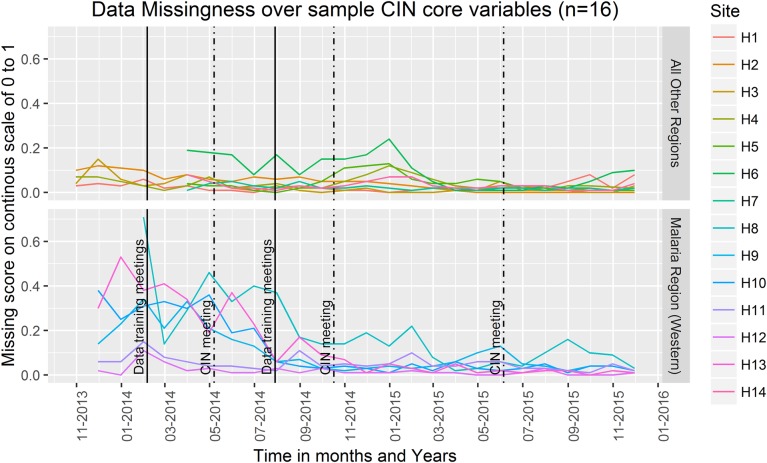
Trends of rate of missing data for signs and symptoms documented during admission included in feedback reports to hospitals. CIN, Clinical Information Network.

To illustrate the overall effect, we created an index of missing data based on the 49 required core admission variables (demographic, symptom and sign characteristics) for each case. A similar index was created for the subset of 16 core clinical characteristics specifically included in the feedback reports. We show in figures 2 and 3 below how clinical documentation has improved (missing data have declined) for each hospital over time, including in these figures an indication of the timing of major CIN meetings. With the improvement in data, fuller descriptions of patient populations are now possible and are presented elsewhere.^28^

**Figure 4:**
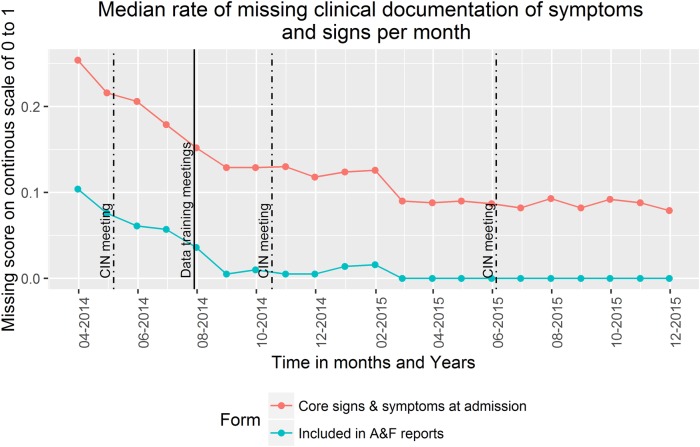
Median rate per month of missing data comparing documentation of items included in feedback reports versus all signs and symptoms collected at admission. CIN, Clinical Information Network.

Such feedback reports and participation in the network have prompted greater adoption and use of the standard paediatric admission record form and, consequently, overall improvements in documentation of clinical characteristics. Plotting the median value of the missing data index for each case record for the broad set of demographic and clinical characteristics and the core set of clinical features suggests that those items that are directly the subject of feedback have shown greater improvement, although there is improvement for all aspects of documentation (figure 4). In a specific example, the recording of the presence or absence of the Alert, Verbal response, Pain, Unresponsive (AVPU) danger signs and ability to drink has improved from 64% in all admissions in the first 3 months each hospital joined the network to 95% in the most recent 3-month period.

## Discussion

### A community of practice

The CIN has been relatively successful in creating an opportunity for frontline caregivers, health researchers and informatics specialists to learn as a community to improve availability of clinical data and begin to promote their use. The hospitals in the network have begun supplying and promoting the use of more structured medical records. This has been helped, we believe, by slowly changing the hospital culture through sustained engagement and by providing peer support by linking hospitals within the network.^9^ ^37^ In this way, new staff quickly become familiar with the clinical forms and are integrated into thinking about data-informed quality improvement efforts at the hospital level, something that is not routine.^37^ ^38^ This is especially important in low-income countries as clinical staff in training programmes (who often are the ones admitting patients) rotate through different hospitals on a 3-monthly basis.

Clinical teams may feel criticised if key indicators show poorer performance than they had been anticipating. The efforts made to adopt an inclusive, facilitative and supportive way of using data have resulted in refinements to indicators that better reflect practice and have built trust in the results. A supportive rather than regulatory approach that appreciates challenges to improvement efforts (eg, lack of Mid-Upper Arm Circumference (MUAC) tapes or pulse oximeters), coupled with face-to-face meetings, is encouraging growing ownership of the data by the clinical teams.

### Digital architecture and links to quality improvement

The CIN collated anonymised data on over 65 000 admissions in its first 2 years of operations. It is producing comprehensive clinical paediatric data, which are of moderately good quality and are trackable. This provides opportunities for exploring the use and value of these data as part of CIN's longer term aims to improve care. A full account of CIN's data management framework is provided elsewhere,^35^ but the focus on using non-commercial or open-source software provides future opportunities for sharing all tools, standard operating procedures and approaches to analysis. At each hospital, only one personal computer, an internet link and a clerk are required, supported by a centralised data management and analysis team working with paediatricians.

The data sharing approach and work to automate production of CIN routine reports means they can be fed back to the hospital management and clinical teams in CIN hospitals as documents and presentations with discussion facilitated by telephone, social networks and occasional face-to-face meetings of network partners. The focus can be put on key indicators that show poor performance in the hospitals and possible interventions suggested, implemented and tested to try to improve clinical performance. For example, the continuing poor documentation of ‘ability to drink’ prompted an exploration of why this occurred in some hospitals when in other hospitals it had improved. A lack of recognition of the value of this sign and limited local supervisory attention were identified as contributory factors.

### Promoting learning

The aim of the CIN is to evaluate common clinical practices and to support the local team take on the responsibility of developing strategies for tackling any deficiencies based on an understanding of the specific hospital context.^5^ The approach thus draws on principles that underlie successful improvement collaboratives. Such collaboratives require data, the primary focus of our initial work. However, the CIN could also support broader learning aims outside the immediate network if a common data framework was adopted across hospitals. This would allow variability in and associations with mortality to be examined and more detailed audit approaches to be added as have been successful in South Africa.^13^ Potentially, such data might be used to track adoption of interventions and their effects over time at scale. One example would be examining diarrhoea/dehydration admissions after introduction of rotavirus vaccination. More specifically, organised networks may contribute to the more efficient conduct of pragmatic trials.^39^ This could help reduce the duration and costs and help enable more rapid translation of research into practice. In other areas, work within the CIN could explore different theory-driven feedback approaches to determine which might be best used to change behaviour. All such learning can feed in at policy level to help develop wider monitoring and evaluation linked to efforts to improve quality and health information systems.

## Conclusions

The work undertaken to date within the CIN has demonstrated that although electronic medical records spanning inpatient care are yet to be deployed in Kenyan wards, it is possible to produce standardised data from multiple sites and improve their quality through partnerships with hospital teams. This has been achieved using low-cost software and innovative adaptations by a local but centralised informatics team working closely with clinicians. These data are used to create timely reports for hospitals that have traditionally had no access to routine information that includes process and outcomes for their patients. Having established this platform, the CIN is now able to begin work with all partners to improve the quality of care and to develop an appreciation of the importance of good information and longer term learning strategies.
