# Correction: *Improving documentation of clinical care within a clinical information network: an essential initial step in efforts to understand and improve care in Kenyan hospitals*

**DOI:** 10.1136/bmjgh-2016-000028corr1

**Published:** 2016-06-03

Tuti T, Bitok M, Malla L, *et al.* Improving documentation of clinical care within a clinical information network: an essential initial step in efforts to understand and improve care in Kenyan hospitals. *BMJ Global Health* 2016;1:e000028. This article has been corrected since it was first published; the abstract has been included.

